# Aeromedical transport of patients receiving extracorporeal membrane oxygenation: a 12-year experience from a regional mobile ECMO network in the French Caribbean

**DOI:** 10.1016/j.aicoj.2026.100148

**Published:** 2026-09-03

**Authors:** Florian Negrello, Tanous Aoun, Alexis Fremery, Camille Almeras, Astrid Monfort, Rishika Banydeen, François Roques, Dabor Resiere, Marc Licker, Papa Gueye

**Affiliations:** a Department of Emergency Medicine, University Hospital of Martinique, 97200 Fort-de-France, Martinique, France; b Cardiovascular Research Team (UR5_3 PC2E), French West Indies University, 97200 Fort de France, Martinique, France; c Department of Cardiac Surgery and Anesthesiology, University Hospital of Martinique, 97200 Fort-de-France, Martinique, France; d Department of Emergency Medicine, University Hospital of French Guiana, 97300 Cayenne, French Guiana, France; e Clinical Investigation Centre - INSERM 1424, University Hospital of French Guiana, 97300 Cayenne, French Guiana, France; f Department of Cardiology, University Hospital of Martinique, 97200 Fort-de-France, Martinique, France; g Department of Critical Care Medicine, University Hospital of Martinique, 97200 Fort-de-France, Martinique, France

**Keywords:** Extracorporeal membrane oxygenation, Mobile health units, Patient transfer, Emergency medical service, Critical care.

## Abstract

**Background:**

The use of extracorporeal Membrane Oxygenation (ECMO) increases for patients with refractory cardiac and/or respiratory failure. To ensure broader access to this life-saving therapy, regional ECMO networks have been developed worldwide, enabling the transfer of patients supported with ECMO to specialized referral centers. At the University Hospital of Martinique, a Mobile Circulatory Support Unit provides 24 h/7 coverage for inter-hospital aeromedical transport of patients receiving ECMO across the French Departments of the America and to mainland France. This study aimed to describe the characteristics, transport modalities, and transport-related adverse events of patients undergoing ECMO-supported aeromedical transfer within this regional mobile ECMO network, as well as their short-term and in-hospital outcomes.

**Methods:**

We conducted a retrospective, observational, single-centre study including patients who underwent ECMO-supported aeromedical transfer coordinated by the Mobile Circulatory Support Unit and Emergency Medical Service between January 2010 and December 2021. We collected data on patient characteristics, ECMO configuration and indication, transport modalities, transport-related adverse events, and short-term and in-hospital outcomes.

**Results:**

Of the 520 patients supported with ECMO during the study period, only the 126 who underwent aeromedical evacuation were included in the analysis, including 87 intra-Caribbean and 39 transatlantic transfers. Patients were predominantly male (61.1%), with a mean age of 43 [29.25−56] years, and at least one comorbidity present in 60.3% of cases. Veno-venous ECMO was predominantly used for intra-Caribbean transfers (80.5%), mainly for acute respiratory distress syndrome, whereas veno-arterial ECMO for refractory cardiogenic shock predominated in transatlantic transfers (87.2%). In-transfer complications occurred in 18.3% of cases and were mainly related to hemodynamic instability, with one transport-related death reported. Overall in-hospital mortality was 54.7%, with no significant difference between intra-Caribbean and transatlantic patients.

**Conclusion:**

Aeromedical transport of critically-ill patients under ECMO support appears feasible and relatively safe in our experience, with a low incidence of serious adverse events. The high in-hospital mortality reflects the acute cardiac and/or pulmonary failure, as well as the burden of comorbidities in this population. Prospective studies using standardized adverse-event collection are needed to more accurately evaluate transport-related risks and longer-term outcomes in this high-risk population.

## Background

Extracorporeal Membrane Oxygenation (ECMO) provides temporary respiratory and/or circulatory, configuring either in a veno-venous (VV ECMO) or veno-arterial (VA ECMO), depending on the clinical indication [1]. Recent technological advances have enabled the development of more compact and portable ECMO systems, making interhospital transport of critically ill patients requiring advanced circulatory or respiratory support feasible [2]. However, portability and suitability for aeromedical transport vary between devices, and dedicated transport-compatible ECMO systems are required. Current indications include refractory cardiac arrest [3], refractory cardiogenic shock [4], and severe acute respiratory failure [5], particularly in the context of acute respiratory distress syndrome (ARDS).

Technological advancements and the integration of ECMO into international guidelines have contributed to its wider adoption. Setting up a mobile ECMO program requires close cooperation between medical centers within a geographical region and a well trained on-call transport service with the ability to assess critically-ill patients, cannulate or initiate ECMO on-site, and transfer the patient to a referral cardiac center for further treatment [6].

**Fig. 1:**
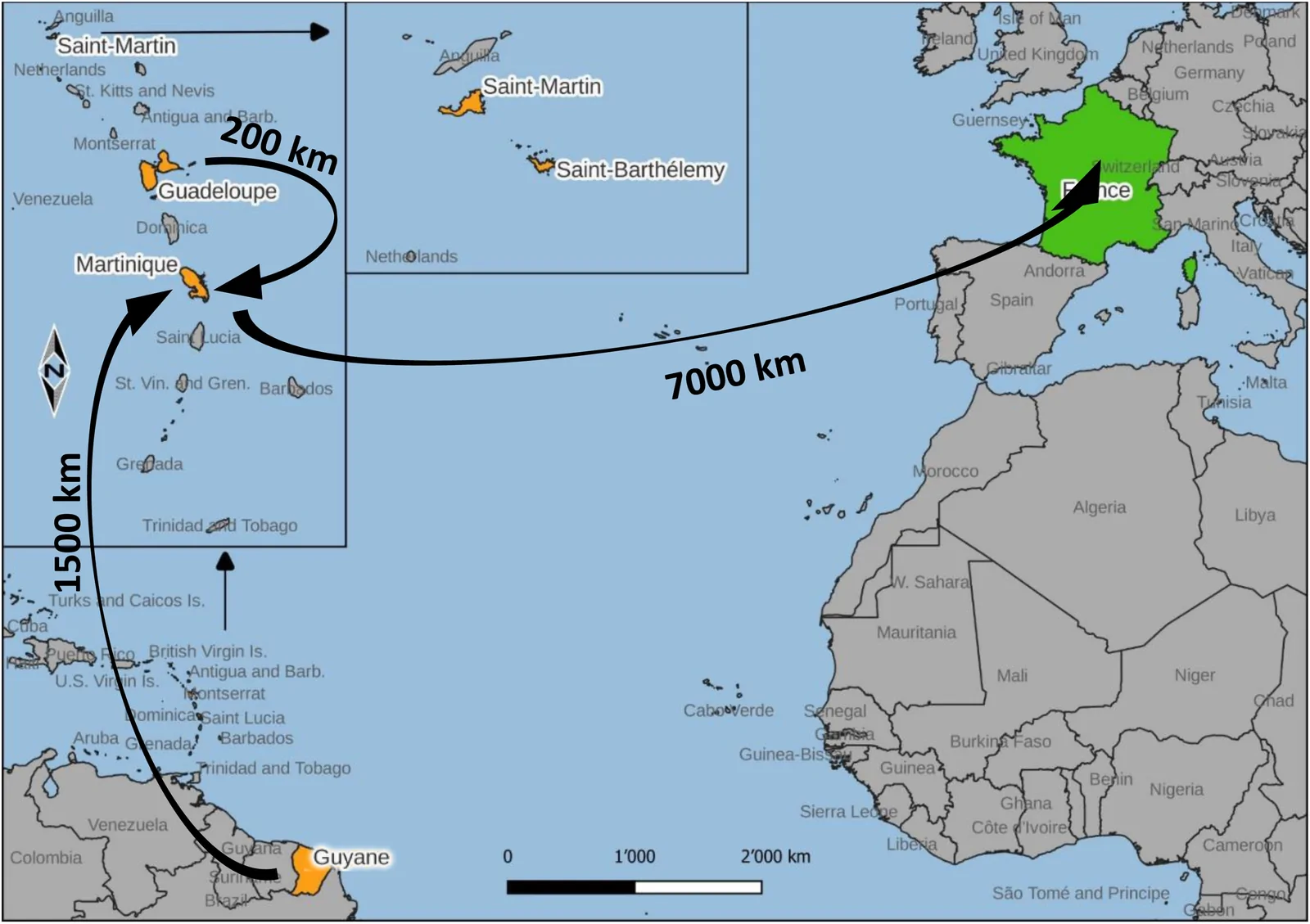
Map of the Eastern Caribbean showing distance between the French department and Martinique, as well as between Martinique and France.

The Heart Team, comprising specialists from the Departments of Cardiology, Cardiac Surgery, and Intensive Care at the University Hospital of Martinique (UHM), provides the full spectrum of therapeutic options for patients with acute or end-stage cardiopulmonary failure, with the exception of heart and lung transplantation. Since 2010, a Mobile Circulatory Support Unit (MCSU) was established to provide ECMO accessibility across the French overseas departments of America, which include the French West Indies (Martinique, Guadeloupe, Saint-Martin and Saint-Barthélémy) as well as French Guiana. This region comprises more than one million inhabitants spread across an area exceeding 85,000 km^2^. Given the geographic constraints of this archipelago and the isolation of French Guiana in South America, separated by distances of up to 1,500 km, aeromedical transport is the only feasible option for interhospital transfer. In some cases, patients may require evacuation to a tertiary referral center in Mainland France, approximately 7,000 km away (Fig. 1). According to the hub-and-spoke model, the Heart Team at the UHM serves as the local referral treatment hub, while the Emergency Medical Services (EMS) coordinate the mobile transport program.

Given the critical condition of these patients, ECMO is initiated on-site by the MCSU team, followed by an aeromedical evacuation to Martinique, coordinated by the EMS of Martinique. A preliminary feasibility study conducted during the first year of activity of the MCSU provided initial data on the feasibility and transport-related adverse events associated with the safety and practicability of this strategy [2]. *Lebreton et al.* emphasized the challenges of such transfers between the UHM and the Pitié-Salpêtrière Hospital of Paris and highlighted a 1-year survival rate of 38.5% in a cohort of 26 patients [7]. To date, data on transport-related adverse events and outcomes in larger cohorts undergoing ECMO-supported aeromedical transfer remain limited.

The primary objective of this study was describe the characteristics, transport-related complications, and early clinical outcomes of patients managed within the regional hub-and-spoke ECMO program. Secondary objectives were to assess short-term mortality and safety of aeromedical transport under ECMO from the French overseas territories of the Lesser Antilles and French Guiana to Martinique, as well as from Martinique to mainland France.

## Methods

### Study design and population

This was a retrospective, observational, single-center study. All consecutive patients who underwent ECMO implantation between January 1, 2010, and December 31, 2021, and who were subsequently air-transferred under ECMO either within the French Caribbean or to mainland France, were included. The MCSU is a dedicated retrieval team responsible for ECMO implantation and/or transport. Depending on the clinical scenario, ECMO was implanted either at the referring hospital during interhospital retrieval missions within the French Caribbean or at the University Hospital of Martinique before secondary aeromedical transfer to mainland France. In both situations, ECMO implantation and patient transport were performed by the same multidisciplinary MCSU team. Eligible cases were identified through EMS and MCSU databases.

### Equipment and transport management

The initial objectives of the MCSU were to perform all on-site VA or VV ECMO implantations throughout the French West Indies and French Guiana and to ensure the safe aeromedical transport of these critically ill patients from remote areas to the referral expert intensive care units at the UHM or mainland France. Indications for VA ECMO and VV ECMO implantation were determined according to current national and international guidelines applicable during the study period. The decision to implantation ECMO was made through a multidisciplinary discussion involving the referring team, mostly the general intensive care unit or cardiac intensive care unit physicians, and the cardiac surgeons and cardiac intensivists of the expert cardiovascular team [6,8]. All transports were performed using the CARDIOHELP-i system (Getinge, Gothenburg, Sweden). The device was secured using its protective and fixation system, and an emergency hand drive was continuously available. It was installed using an aviation-approved fixation system compatible with the aircraft used by our EMS. The escort team comprised a cardiac surgeon, a perfusionist nurse, and an intensivist or emergency physician, with the additional participation - particularly during transatlantic transfers or when space allowed - of a critical care nurse. Transport time was defined as the interval between disconnection from the referring hospital's oxygen and power supplies and reconnection to the receiving hospital's oxygen and power supplies. Aeromedical transport resources were selected according to distance and operational requirements and included military aircraft, civil protection helicopters, private aviation, and commercial airlines, particularly for transatlantic transfers

### Data collection

Data were extracted from EMS dispatch records, MCSU logs, prehospital care unit reports, and hospital medical charts. Collected variables included sociodemographic characteristics, clinical presentation, indication for ECMO support, type of ECMO (VA or VV ECMO), transport-related complications defined as adverse events occurring exclusively during patient transport, from departure from the referring hospital until arrival at the receiving center, and early mortality within 48 h.

### Outcomes

Complications occurring during aeromedical evacuation, early 48 -h mortality and hospital mortality were screened and analyzed. Transport-related complications were identified retrospectively through a systematic review of the standardized transport reports completed by the retrieval team, together with the referring and receiving hospital medical records. Complications associated with medical air transportation were categorized as related to the patient’s condition, staffing and equipment failure. Transport-related complications were classified according to the risk categories [9]. When multiple adverse events occurred during the same transport, only the complication considered to have the greatest clinical impact was recorded in the study database. Death occurring during transport was categorized as a Risk Category I event, reflecting the highest level of severity.

### Statistical analysis

Categorical variables are presented as count with percentages. The distribution of continuous variables was assessed using the Shapiro-Wilk test. Continuous variables are presented as median and interquartile ranges. Continuous variables for which the normality assumption was not rejected are compared between the two independent groups using Student’s t-test. Non-normally distributed continuous variables are compared using the Mann-Whitney U test. Categorical variables were compared using Pearson’s chi-square test or Fisher’s exact test when expected cell counts were small. All tests were two-sided, and a P-value <0.05 was considered statistically significant. Analyses were performed using SAS software (version 9.4; SAS Institute, Cary, NC).

### Ethics

The study was conducted in accordance with the Declaration of Helsinki and applicable French laws. Ethical approval was obtained from the institutional review board of the UHM with waived individual patient consent (IRB Approval No. 2022/161). The extracted data were analyzed anonymously.

## Results

**Fig. 2:**
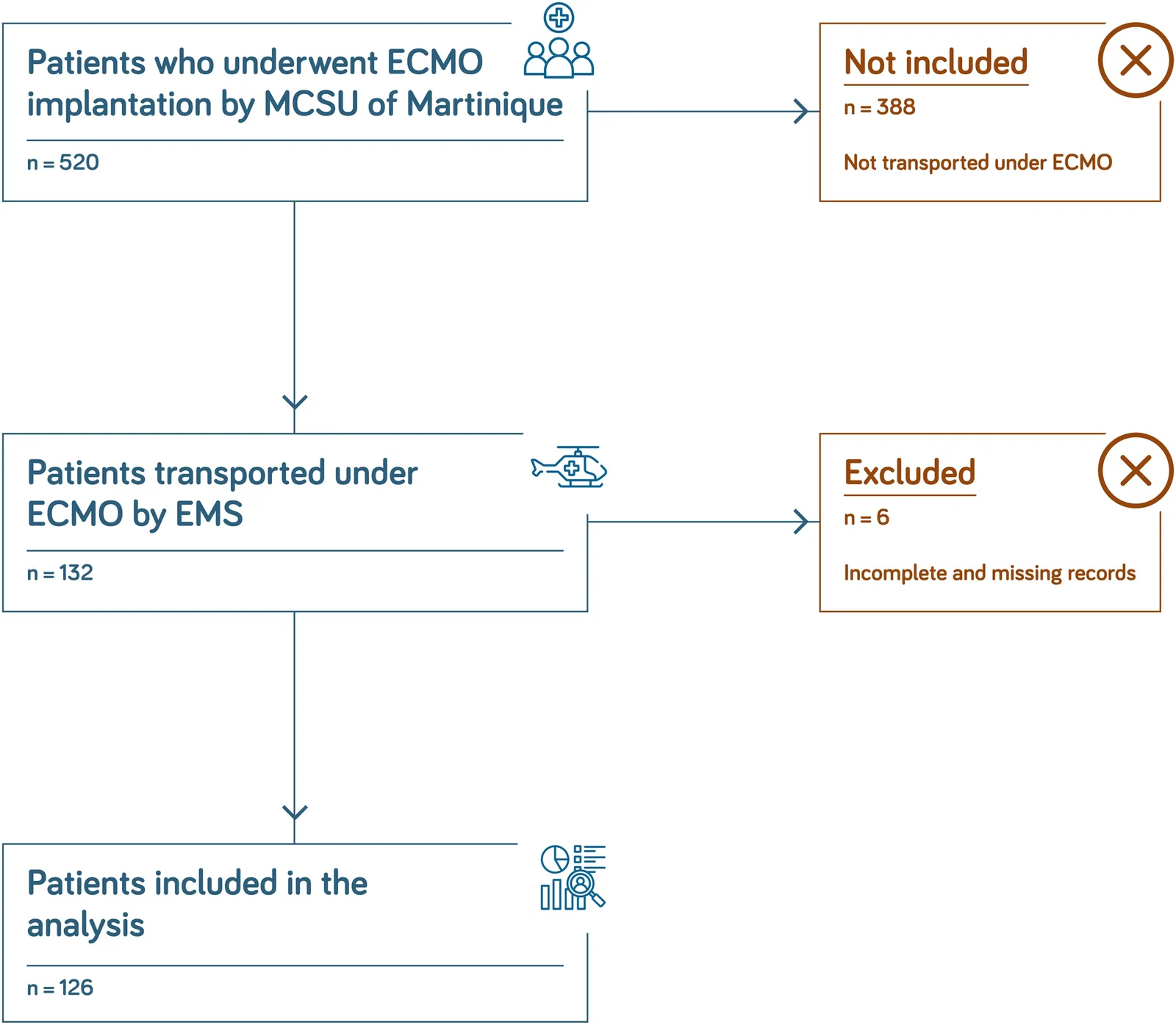
Flow chart. Abbreviations: ECMO (Extra-Corporeal Membrane Oxygenation); EMS (Emergency Medical Service); MCSU (Mobile Circulatory Support Unit)

Between January 1, 2010, and December 31, 2021, the MCSU from the UHM performed a total of 520 ECMO implantations. After exclusion of 394 patients, complete data were available for 126 patients who were included in the final analysis (Fig. 2). Among them, 51 patients (40.5%) received VA ECMO support and 75 (59.5%) received VV ECMO.

A median 11 [[7], [8], [9], [10], [11], [12], [13]] ECMO-related aeromedical evacuations per year were performed in joint collaboration between the MCSU and the EMS of Martinique, Guadeloupe and French Guiana. The first regional air-transport from Guadeloupe to Martinique was carried out in 2010, and the first transatlantic transfer between Martinique and mainland France took place in 2011. Patients were predominantly male (61%), with a median age of 43 [29,25−56] years and comorbidities were present in 60% of cases (Table 1).

**Table 1:** Characteristics of patients undergoing aeromedical transfers with extracorporeal support (2010–2021).

	Intra-Caribbean aeromedical evacuation (n = 87)	Transatlantic aeromedical evacuation (n = 39)	Total (N = 126)	P-value
**Age (years),***median [IQR]*	43 [33−56]	41 [27.5−57]	43 [29,25−56]	0.647
**Male sex,***n (%)*	52 (59.8)	25 (64.1)	77 (61.1)	0.645
**Comorbidities,***n (%)*				
None	43 (49.4)	7 (18.0)	50 (40.5)	**0.001**
Heart failure with LVEF < 50%	6 (6.9)	19 (48.7)	25 (19.8)	**<0.001**
Venous thromboembolism	4 (4.6)	2 (5.1)	6 (4.8)	1.000
Arterial hypertension	23 (26.4)	10 (25.6)	33 (26.2)	0.925
Hematologic disease^1^	2 (2.3)	0 (0.0)	2 (1.6)	1.000
Chronic respiratory failure^2^	6 (6.9)	2 (5.1)	8 (6.4)	1.000
Cancer	4 (4.6)	3 (7.7)	7 (5.6)	0.676
Chronic kidney disease^3^	5 (5.8)	3 (7.7)	8 (6.4)	0.702
Diabetes	14 (16.1)	4 (10.3)	18 (14.3)	0.387
Tobacco/alcohol use	17 (19.5)	15 (38.5)	32 (25.4)	**0.024**
**Type of ECMO,***n (%)*				**<0.001**
VA ECMO	17 (19.5)	34 (87.2)	51 (40.5)	
VV ECMO	70 (80.5)	5 (12.8)	75 (59.5)	
**Indication of ECMO,***n (%)*				**<0.001**
Post-cardiac arrest^4^	1 (1.2)	3 (7.7)	4 (3.2)	
Primary cardiogenic shock^5^	7 (8.1)	15 (38.5)	22 (17.5)	
Secondary cardiogenic shock^6^	3 (3.5)	16 (41.0)	19 (15.1)	
Obstructive cardiogenic shock	2 (2.3)	0 (0.0)	2 (1.6)	
Cardiogenic shock due to cardiotoxic agents	1 (1.2)	0 (0.0)	1 (0.8)	
Community-acquired infectious ARDS^7^	29 (33.3)	3 (7.7)	32 (25.4)	
Nosocomial infectious ARDS^8^	16 (18.4)	0 (0.0)	16 (12.7)	
COVID-19-related ARDS	21 (24.1)	1 (2.6)	22 (17.5)	
Non-infectious ARDS^9^	7 (8.1)	1 (2.6)	8 (6.4)	

Abbreviations: ARDS (Acute Respiratory Distress Syndrom); ECMO (Extra-Corporeal Membrane Oxygenation); LVEF (Left Ventricular Ejection Fraction); VA (Veno-Arterial); VV (Veno-Venous).

1 Sickle cell disease, hemophilia.

2 Any chronic respiratory disease requiring long-term therapy and/or equipment.

3 Known glomerular filtration rate <60 mL/min/1.73 m^2^ before hospitalization.

4 Cardiogenic shock following recovered cardiac arrest.

5 First manifestation of undiagnosed cardiomyopathy.

6 Decompensation of pre-existing known cardiomyopathy.

7 Onset <48 h after hospital admission, non-COVID-19.

8 Onset >48 h after hospital admission, non-COVID-19.

9 Occurring in context of non-infectious systemic inflammatory response syndrome.

Regional evacuations within the French Departments of the America accounted for 69% of cases (n = 87). Transport modalities included helicopters (84%), commercial aircrafts (8%), military aircrafts (5%), and private jets (3%). The flight durations varied according to both the mode of transport and then departure location, averaging approximately 1 h from Guadeloupe (73 transfers) or Sainte Lucia (1 transfer), and 4 h from French Guiana (13 transfers). Transatlantic evacuations represented 31 % of cases (n = 39), all performed using commercial aircrafts, with an average flight duration of 8 h and 30 min. Among these 39 patients transferred to mainland France, 9 had previously undergone an initial regional aeromedical transfer from a remote island or from the French Guiana to the UHM. All intra-Caribbean transfers were performed on the same day as ECMO implantation, whereas patients transferred to mainland France were transported a median of 2 [1–4.5] days after ECMO implantation.

**Fig. 3:**
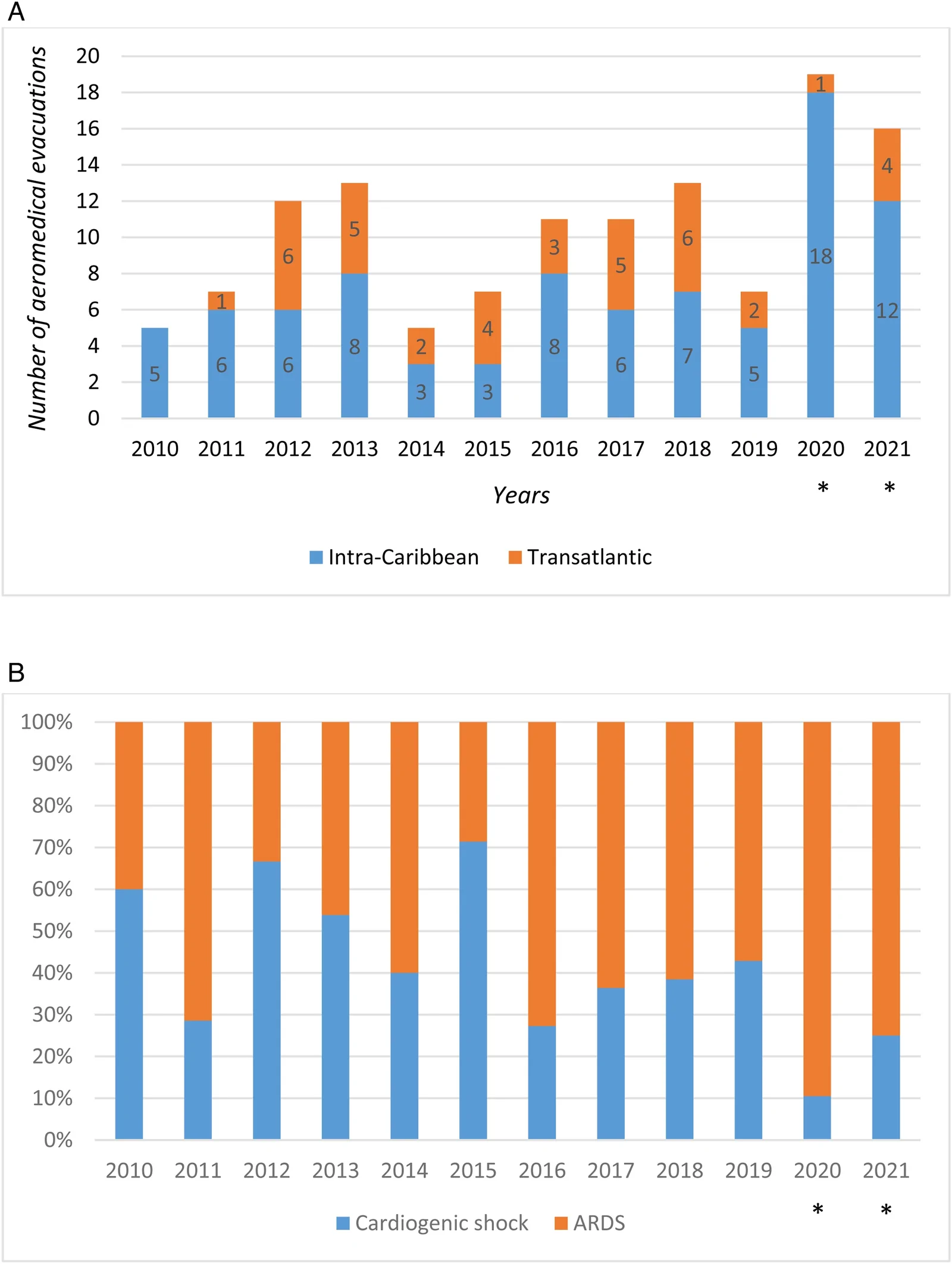
(A) Number of aeromedical evacuation under Extracorporeal Membrane Oxygenation between 2010 and 2021. (B) Indications of Extracorporeal Membrane Oxygenation requiring aeromedical evacuations between 2010 and 2021. *Indicate the years affected by the COVID-19 pandemic.

Patients transferred from the UHM to mainland France were predominantly with VA ECMO support (87%, n = 34), mainly for cardiogenic shock, whereas those transported from Caribbean area to the UHM were predominantly with VV ECMO (81%, n = 70), primarily for severe ARDS (Table 1). The temporal evolution of these medical evacuation is illustrated in Fig. 3A and 3B.

Transfer complications occurred in 18% of cases. According to the Fletcher-Sandersjöö risk classification, 1 event (4.3%) was classified as Risk Category I (1 death during transport), 19 events (82.6%) as Risk Category II (8 cardiac arrhythmias, 2 pulmonary edemas, 8 clinical signs of shock, and 1 cannulation-site bleeding), and 3 events (13.0%) as Risk Category III (3 acute limb ischemia). No Risk Category IV events were observed (Table 2). No complication related to the staffing or equipment failure was identified. Complications were more frequently observed during transatlantic transfers than during regional transfers (p-value < 0.001).

**Table 2:** Outcomes and mortality of patients undergoing aeromedical extracorporeal Membrane Oxygenation transfers (2010–2021).

	Intra-Caribbean aeromedical evacuation (n = 87)	Transatlantic aeromedical evacuation (n = 39)	Total (N = 126)	P-value
**Complications during transport**, *n (%)*	9 (10.3)	14 (35.9)	23 (18.3)	**<0.001**
Risk category I	1 (1.1)	0 (0.0)	1 (0.8)	
Risk category II	7 (8.0)	12 (30.8)	19 (15.1)	
Risk category III	1 (1.1)	2 (5.1)	3 (2.4)	
Risk category IV	0 (0.0)	0 (0.0)	0 (0.0)	
**Post-transfer ECMO duration (days),***median [IQR]*	9 [4–15]	15 [11.5−21]	115 [5−18.75]	**<0.001**
**Surgical procedure**, *n (%)*	3 (3.4)	22 (56.4)	25 (19.8)	**<0.001**
Cardioverter-defibrillator placement	0 (0)	1 (2.6)	1 (0.8)	
Cardiac valve replacement	2 (2.2)	1 (2.6)	3 (2.4)	
Neonatal congenital heart surgery	0 (0.0)	1 (2.6)	1 (0.8)	
Central Veno-Arterial ECMO	0 (0.0)	1 (2.6)	1 (0.8)	
Left ventricular assist device implantation	0 (0.0)	7 (17.9)	7 (5.6)	
Heart transplantation	0 (0.0)	9 (23.1)	9 (7.1)	
Total artificial heart implantation	0 (0.0)	2 (5.1)	2 (1.6)	
Pulmonary surgical procedure	1 (1.1)	0 (0)	1 (0.8)	
**Mortality in the receiving ICU**, *n (%)*	40 (46.0)	21 (53.8)	61 (48.4)	0.41
Mortality < 48 h post-transfer	9 (10.3)	2 (5.1)	11 (8.7)	0.34
By ECMO indication				
Post-cardiac arrest^1^	1 (1.1)	2 (9.5)	3 (2.4)	
Primary cardiogenic shock^2^	1 (1.1)	9 (23.1)	10 (7.9)	
Secondary cardiogenic shock^3^	1 (1.1)	9 (23.1)	10 (7.9)	
Obstructive cardiogenic shock	0 (0.0)	0 (0.0)	0 (0.0)	
Cardiogenic shock due to cardiotoxic agents	0 (0.0)	0 (0.0)	0 (0.0)	
Community-acquired infectious ARDS^4^	15 (17.2)	1 (2.6)	15 (11.9)	
Nosocomial infectious ARDS^5^	5 (5.7)	0 (0.0)	5 (4.0)	
COVID-19-related ARDS	3 (3.4)	1 (2.6)	4 (3.2)	
Non-infectious ARDS^6^	14 (16.1)	0 (0.0)	14 (11.1)	
**In-hospital mortality**, *n (%)*	46 (52.9)	23 (59.0)	69 (54.8)	0.52

Abbreviations: ARDS (Acute Respiratory Distress Syndrome); ECMO (Extra-Corporeal Membrane Oxygenation); ICU (Intensive Care Unit).

1 Cardiogenic shock following resuscitated cardiac arrest.

2 First manifestation of undiagnosed cardiomyopathy.

3 Decompensation of pre-existing cardiomyopathy.

4 Onset < 48 h after hospital admission, non-COVID-19 related.

5 Onset > 48 h after hospital admission, non-COVID-19 related.

6 Occurring in the context of non-infectious systemic inflammatory response syndrome.

Patients managed at the UHM following Caribbean transfer underwent few surgical procedures, consistent with the pulmonary etiology underlying most ECMO indications (Table 2). Only two valve replacements and one lobectomy were performed. In contrast, patients transferred to reference centers in mainland France underwent advanced cardiac surgical interventions in 56% of cases (Table 2), including cyanotic congenital heart surgery (n = 1), cardiac valve replacement (n = 1), implantable cardioverter-defibrillator placement (n = 1), left ventricular assist device implantations (n = 7), total artificial heart implantation (n = 2), and heart transplants (n = 9).

Overall, 69 patients (54.7%) died during their hospitalization following ECMO-supported aeromedical transfer, including 11 patients (9%) within the first 48 h after the transport, without significant difference between intra-Caribbean and transatlantic transfers (Table 2). Mortality was similar in patients supported for ARDS (49%) and those supported for cardiogenic shock (48%). Reported causes of death were refractory ARDS (33%), septic shock (28%), refractory cardiogenic shock (15%), cardiac arrest (10%), hemorrhagic shock (7%), intracranial hemorrhage (5%), reperfusion syndrome (2%), and severe drug-induced skin reaction (2%).

## Discussion

This retrospective study describes the characteristics, transport modalities, transport-related adverse events, and hospital outcomes of 126 patients who underwent ECMO-supported aeromedical transfer within a regional mobile ECMO network between 2010 and 2021. Approximately two-thirds of the patients underwent intra-Caribbean transfer to the UHM, the regional referral centre for cardiovascular surgery and extracorporeal circulatory support, whereas approximately one-third underwent transatlantic transfer from Martinique to mainland France. Transport-related adverse events were documented in 18.3% of cases, and overall in-hospital mortality was 54.7%. The two transfer groups represented distinct clinical pathways: intra-Caribbean transfers predominantly involved patients receiving VV ECMO for severe respiratory failure, whereas transatlantic transfers predominantly involved patients receiving VA ECMO to access advanced cardiac therapies unavailable in the French Caribbean.

Despite the highly specific context of the EMS of Martinique experience, this activity remains poorly reported. Existing publications are limited to small case series, predominantly describing short-range transfers, and rarely by aeromedical transport [[9], [10], [11], [12], [13], [14]]. Nevertheless, ECMO transport appears feasible and safe, although most available data are retrospective and share similar limitations with our study, notably possible underreporting of in-flight complications and lack of long-term outcomes. In this cohort, no transport-related adverse event was documented in 81.7% of cases. When complications did occur, they were rarely immediately life-threatening. Only one fatal event was reported during transport, involving a patient with newly initiated ECMO for refractory ARDS due to pneumonia. These findings document the feasibility of ECMO-supported aeromedical transfer within this geographically dispersed regional setting, consistent with smaller previously published series [2,10,15]. However, because of the retrospective design, the absence of a comparator group, and the possibility of underreporting of adverse events, they should not be interpreted as demonstrating the safety or effectiveness of the transfer strategy. Available data on complications related to ECMO in the literature mainly concern cannulation and decannulation-related events and bleeding-associated, which are associated with a very poor prognosis when they occur in hospitalized populations, up to 23% cases resulted in death [16].

It is important to emphasize that the actual duration of patient management by the EMS extends far beyond flight time and therefore represents a substantially longer period of exposure to potential transport-related complications [9,17]. In addition to the time required for ECMO preparation and cannulation, inter-facility transfers to and from airports or helipads and multiple patient handlings significantly prolong management. Total management time is particularly extended during transatlantic missions on commercial flights. The transatlantic transfer itself, from the referring hospital to the receiving hospital, including inter-facility ground transportation before departure and after landing, could last up to 12 h. When the time required for patient assessment, stabilization, and transport preparation was added, the overall duration of patient management was substantially longer. This prolonged exposure time may partly explain the higher complication rate observed during transatlantic transfers, which involve more complex logistics, extended exposure to critical care conditions and environmental stressors such as low humidity, hypobaric pressure, vibration, or cold temperatures at high-altitude flight [18,19]. However, this association cannot be isolated from major differences in ECMO configuration, underlying indication, clinical severity, therapeutic objectives, and transport logistics between the two pathways. The proportion of transfers with documented adverse events in our cohort was 18.3%, compared with rates ranging from 19% to 37% in previous case series. However, these figures should be compared cautiously because adverse-event definitions, ascertainment methods, transport conditions, and patient populations differed across studies [13,20,21].

The COVID-19 pandemic had a significant impact on ECMO care delivery, particularly during the period 2020–2022. In Martinique, ECMO was offered to patients with SARS-CoV-2-related ARDS as long as intensive care unit capacity permitted, despite early uncertainty regarding its benefit in advanced disease [22]. Conversely, the number of patients supported with ECMO for cardiogenic shock or non-COVID-related ARDS declined during successive pandemic waves. Transatlantic transfers were also significantly affected: in 2020, missions were temporarily suspended due to intensive care unit saturation in mainland France, restricting access to advanced cardiac therapies. These constraints highlight the vulnerability of ECMO networks during global health crises and the need for adaptive strategies to maintain access to life-sustaining therapies in geographically isolated territories.

Among all patients who underwent ECMO-supported aeromedical evacuation, initial intensive care unit mortality was 48 % and in-hospital mortality 55%. These mortality rates were observed in a heterogeneous population with severe refractory cardiac or respiratory failure. However, the available data do not allow the respective contributions of baseline disease severity, patient selection, ECMO-related factors, and transport-related factors to be determined [[23], [24], [25], [26], [27]]. Further studies are warranted to evaluate long-term outcomes, patient trajectories, and the cost-effectiveness of such resource-intensive interventions.

### Limitation

This study has several limitations. First, its retrospective and single-center design exposes it to inherent limitations that reduce the generalizability of the findings to other settings with different organizational structures or resources. Because of the retrospective design, we cannot ensure that all transport-related complications were consistently documented in the medical records. Consequently, the overall burden of transport-related complications may have been underestimated.

Second, the study cohort comprised only patients who underwent ECMO-supported aeromedical transfer. It did not capture the entire population potentially considered for mobile ECMO retrieval, including patients who may have been referred but not cannulated, as well as patients who may have been cannulated but were not subsequently transported. Consequently, the study population represents a selected group of patients who were considered eligible for ECMO and stabilized to proceed to aeromedical transfer. This may have introduced both selection and survivorship biases and limits the representativeness of the cohort with regard to all patients referred to the mobile ECMO team. The findings should therefore not be extrapolated to the entire population potentially eligible for mobile ECMO retrieval.

Third, the sample size may have limited the statistical power to detect differences in outcomes, particularly between intra-Caribbean and transatlantic transfers. Furthermore, the two groups represented fundamentally different clinical pathways and differed substantially in ECMO configuration, underlying indication, therapeutic objective, clinical severity, and transport duration. These differences, together with the heterogeneity of transport modalities and patient characteristics, introduce substantial confounding and preclude attributing the observed differences between groups to transport distance or duration alone. the heterogeneity of indications for ECMO, transport modalities, and clinical severity not systematically available throughout the 12-year study period may have influenced outcomes and limits the ability to draw causal inferences.

Finally, the absence of a control group of non-transported patients receiving ECMO precludes any direct assessment of the effect of aeromedical transfer on patient outcomes. Accordingly, the documented rates of transport-related adverse events and mortality should be interpreted as descriptive findings from a selected cohort and do not establish the safety or effectiveness of the transport strategy or support causal inferences regarding its impact on prognosis.

## Conclusion

This 12-year retrospective study describes ECMO-supported aeromedical transfers conducted within a regional mobile ECMO network serving the French Caribbean and French Guiana. Transport-related adverse events were documented in 18.3% of cases, including one death during transport, while overall in-hospital mortality was 54.7%. These findings support the operational feasibility of ECMO-supported aeromedical transfer in a geographically dispersed region. Prospective studies using standardized patient-selection criteria, severity assessments, and adverse-event definitions are needed to evaluate transport-related risks and longer-term patient outcomes, functional recovery, and quality of life in this high-risk population.

## Author contribution

Florian Negrello: Conceptualisation, Methodology, Formal analysis, Writing - original draft.

Tanous Aoun: Writing - original draft.

Alexis Fremery: Writing - review & editing.

Camille Almeras: Investigation.

Astrid Monfort: Writing - review & editing.

Rishika Banydeen: Writing - review & editing.

Francois Roques: Writing - review & editing.

Dabor Resiere: Writing - review & editing.

Marc Licker: Writing - original draft.

Papa Gueye: Conceptualisation, Writing - original draft.

## Funding

None.

## Declaration of competing interest

No conflict of interest.
